# Discriminatory abilities of facultative slave-making ants and their slaves

**DOI:** 10.1007/s00040-016-0493-z

**Published:** 2016-06-17

**Authors:** T. Włodarczyk

**Affiliations:** Department of Invertebrate Zoology, University of Białystok, Ciołkowskiego St 1 J, 15-245 Białystok, Poland

**Keywords:** Ants, Nestmate recognition, Slave-making, Social parasitism

## Abstract

Intra-colony odor variability can disturb ants’ ability to discriminate against intruders. The evolutionary relevance of this phenomenon can be revealed by studies on colonies of slave-making ants in which the parasite, and not the host, is subject to selection pressures associated with living in a mixed colony. We examined how the European facultative slave-making species *Formica sanguinea* and its *F. fusca* slaves perform in discriminating ants from alien colonies. Results of behavioral assays showed that slave-maker ants respond with hostility to conspecific individuals from alien colonies but are relatively tolerant to alien slaves. Furthermore, the behavior of slaves indicated a limited ability to discriminate ants from alien parasitic colonies. The subdivision of colony fragments into mixed and species-separated groups demonstrated that contact with the parasite is necessary for *F. fusca* slaves to be re-accepted by former nestmates after a period of separation from the stock colony. The results presented in this paper are consistent with the following hypotheses: (1) *F. sanguinea* ants, as opposed to their slaves, are adapted to discriminate alien individuals in the conditions of odor variability found in a mixed-species colony, (2) the recognition of slaves by *F. sanguinea* ants involves a dedicated adaptive mechanism that prevents aggression toward them, (3) the odor of slaves is strongly influenced by the parasite with beneficial effect on the colony integrity.

**Electronic supplementary material:**

The online version of this article (doi:10.1007/s00040-016-0493-z) contains supplementary material, which is available to authorized users.

## Introduction

An insect society can be viewed as a divided organism (Strassmann and Queller 2007), therefore, its efficient functioning relies on cooperation between mobile, discrete parts (colony members), which are equipped with the ability of mutual recognition. The importance of nestmate discrimination becomes apparent in such activities as territory defense, colony relocation or food retrieval and distribution. Information about colony membership is perceived by chemosensory organs and conveyed by a specific mixture of hydrocarbons and lipids covering the insect’s body surface (Zweden and d’Ettorre 2010). Much evidence indicates that in most ant species, common colony odor is achieved through the exchange of cuticular hydrocarbons (CHC) between colony members (Soroker et al. 1995; Dahbi et al. 1999; Boulay 2000; Lenoir et al. 2001). This odor can be represented as a template encoded in each individual’s higher brain centers. An ability for nestmate recognition can also be achieved on the level of the peripheral sensory system by the desensitization of CHC specific receptor neurons to an individual’s own CHC blend, which is constantly transported into the cavity of the chemosensilla located on the antennae (Ozaki and Hefetz 2014). Regardless of the neuronal mechanism, it has been demonstrated that odor learning takes place shortly after pupal eclosion, which does not rule out later updates following a temporal variation in the colony’s odor. This imprinting-like phenomenon can be utilized to form artificial mixed ant colonies from individuals which have just emerged from pupae, even when they belong to different genera or subfamilies (Fielde 1903, 1904; Errard and Jaisson 1991). Mixed colonies are also found in nature as a result of social parasitism. A spectacular example is provided by slavemaking ants, which pillage the pupae of host species and bring them back to their own nest where their development is completed. Callow workers, which have emerged from the robbed pupae, learn to recognize the odor of ants from the parasite colony and, thereafter, treat it as a nestmate recognition label (Errard 1984; Errard and Jaisson 1991; Errard 1994a). In mixed colonies, interspecific differences in the genetic pattern of CHC production may override the effect of the exchange of compounds between nestmates (see Bagnères et al. 1991). This leads to an elevated odor variability within the colony. Thus, mixed colonies provide a convenient experimental opportunity to gain insight into how the variability of reference odors determines the outcome of dissimilarity assessment and subsequent discriminatory behavior.

In artificially formed mixed colonies of *Manica rubida* and *Formica selysi*, both species seem to develop an internal template that includes the odor of an allospecific partner, which is accompanied by a partial adjustment of chemical labels (Errard 1994a, b). However, living in a mixed colony with *Tetramorium caespitum* or *F. selysi* ants causes an elevated frequency of recognition errors by *M. rubida* during exposure to non-nestmates (Errard et al. 2006a). This could be explained by the fact that both *F. selysi* and *T*. *caespitum* have odors remarkably different from those of *M. rubida* (Errard et al. 2006a). An internal template which has been developed under the conditions of a mixed colony might be imprecise, since the process of its formation may be constrained by an evolutionarily-shaped propensity for the species-specific odor.

Although the similarity of the hydrocarbon profiles between host and parasite workers has been observed (Yamaoka 1990; Bonavita-Cougourdan et al. 1996; Brandt et al. 2005; Ruano et al. 2011; Bauer et al. 2010), its role and adaptive value has not been unequivocally explained (see Discussion in Brandt et al. 2005). It could be that a restricted capacity of ants to develop an accurate template of different odors may have promoted evolutionary changes, which resulted in odor similarity between species that frequently meet as mixed colony members in natural conditions. Although this may be the simple effect of hydrocarbon exchange between nestmates, at least in the case of *Polyergus rufescens*, the innate pattern of CHC biosynthesis mimics that of the most frequently used host species (D’Ettorre et al. 2002). Nevertheless, chemical mimicry in slave-making ants is not perfect, since host-parasite differences in hydrocarbon profiles can be detected in their colonies (Liu et al. 2003; Brandt et al. 2005; Errard et al. 2006b). Such differences can be expected also in colonies of the facultative slave-making ant species *Formica sanguinea* given that this social parasite has distinct CHC profile from the primary host species *F. fusca* (Martin et al. 2008). We present here the results of a study on the potential consequences of recognition label differences between host and parasite for performance in the nestmate discrimination in *F. sanguinea* colonies containing *F. fusca* slaves. The parasite’s recognition system may be evolutionarily adapted to manage high odor variability within the colony associated with the presence of heterospecific nestmates. In contrast, no such adaptation is expected of *F. fusca* slaves, since their colonies consist of related individuals (Hannonen and Sundström 2003), hence, colony members could be expected to share common alleles at recognition cue loci leading to relatively low, genetic-based odor variability. It could be expected that exotic social environment with remarkable differences in recognition signatures between colony members may disrupt slaves’ ability to discriminate nestmates. This in turn would negatively affect slave-maker colony due to recognition errors. Consequently, slave-making ants may have evolved a strategy to control this effect. Thus, the following hypotheses could be proposed: (1) *F. fusca* slaves, which are not adapted for functioning under conditions of extraordinary reference odor variability, are too permissive during encounters with non-nestmates (false acceptances) and too intolerant toward nestmates (false rejections), (2) *F. sanguinea* ants are intrinsically tolerant of any individuals with traits indicating that they might be slaves, (3) *F. sanguinea* affects recognition signature of slaves to increase the chances of their efficient integration and functioning in a parasite’s colony. Alternatively for the second hypothesis, slave acceptance by *F. sanguinea* ants is not an inherited response to fixed key-signals but instead is based on an ability to develop a fine-tuned template under conditions of high intra-colony odor variation. They should then adequately discriminate their own slaves from any kind of non-nestmates, including alien slaves. We attempted to find evidence supporting the hypotheses presented above by examining the discriminatory behavior of *F. sanguinea* ants and their slaves during confrontations with ants from alien colonies, as well as with ants from colony fragments split into homospecific and mixed groups.

## Methods

### Laboratory colonies

Ants used for this study were collected from the Knyszyn Forest, N-E Poland (53°11′N, 23°20′E). Eight queenright *Formica sanguinea* colonies were collected between April 8 and May 1, 2014. The proportion of *F. fusca* slaves in these colonies were estimated to fall within a range of from 8 to 57 % (mean = 34 %). Moreover, 8 queenright *F. fusca* colonies and 8 *F. polyctena* colony fragments, including 2 queenright ones, were acquired from the field from March 27 to June 27. The laboratory cultures were maintained in plastic boxes (60 × 45 × 30 cm for *F. sanguinea* and 40 × 30 × 30 cm for *F. fusca* and *F. polyctena*), which had their inner walls coated with liquid paraffin to prevent the ants from escaping. *Formica sanguinea* and *F. fusca* nest boxes had a thin layer of soil and were provided with mosses, while *F. polyctena* boxes were maintained in the material taken from their mounds, supplemented with plant debris removed from the forest floor. All nest boxes were provided with test tubes fitted at one end with a water container. To maintain humidity, nest boxes were moistened regularly with the use of a water sprayer. All colonies were fed with diluted honey, while those containing a queen’s brood were additionally provided with dead crickets (*Acheta domestica*) and honeycomb moth (*Galleria mellonella*) larvae. A natural photoperiod was applied in the rearing room.

For the separation experiment, three queenless daughter colonies were formed from each of the eight slave-maker queenright (stock) colonies: one mixed retaining the original proportion of slaves, and two pure (one per species). At the beginning, these colonies contained between 75 and 415 workers (median = 230), except for one *F. fusca* pure colony, which was established with only 22 workers due to the low overall number of workers in the stock colony coupled with a low slave proportion. Daughter colonies were maintained in smaller plastic boxes (25 × 25 × 12 cm) and the remaining culture conditions were similar to those of the stock colonies. To partly compensate for the lack of a queen, each of the daughter colonies was provided with 5–75 eggs collected from the stock colonies (the number of eggs was similar across the colonies and originating from the same stock colony). The colonies used for separation experiment were formed between May 14 and 23, 2014, 40–79 days prior to the behavioral tests.

### Behavioral assays

The behavior of four ants (evaluators) toward one ant from a different or the same colony (cue-bearer—terminology after Liebert and Starks 2004) was analyzed. The ants were placed in a Petri dish (5.5 cm in diameter) covered with a transparent plastic plate. Each ant was used in only one test. *Formica sanguinea* evaluators were randomly sampled from ants active outside of the nest whereas *F. fusca* ants in this role had to be removed from the nest due to their low external activity (as in natural conditions, see Kharkiv 1997; Mori et al. 2000). The cue-bearer was anesthetized in carbon dioxide before starting the test to reduce the influence of its behavior, especially of aggressive acts, on the experiment’s results. The test began by removing a paper barrier separating the cue-bearer and the evaluators, which was done 2 min after placing the last ant in the Petri dish. Between the tests, the Petri dish and cover plate were cleaned with ethanol and the paper barrier was replaced with a new one. A single bioassay lasted 5 min and was video-recorded at a resolution of 480 × 640 pixels for further analysis. The person who analyzed the video recordings was unaware of the ants’ colony of origin. The occurrence of antennation or aggressive behaviors (mandible spreading, biting, biting with flexed gaster) was determined each 10 s and scored by the number of ants displaying it. After summing up scores across a single test, the frequency of aggressive behaviors or antennation was obtained which could be further totaled to give the frequency of pooled behaviors in that test.

### Experiment design

**Fig. 1:**
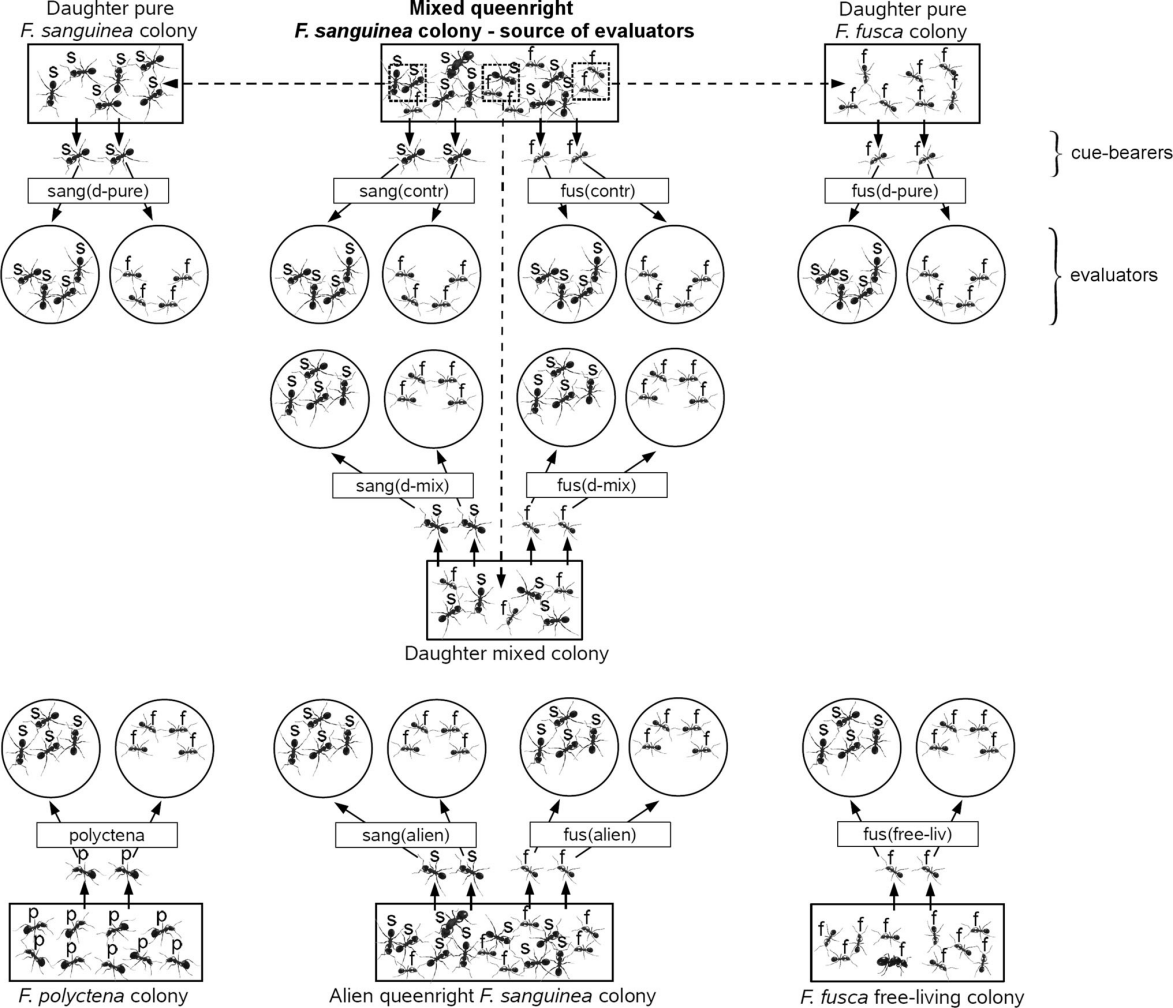
Scheme illustrating the experimental design. Each colony was a donor of cue-bearer placed in Petri dish along with four evaluators from “Mixed queenright *F. sanguinea* colony”. *Abbreviations* in *boxes* indicate treatment category applied in two variants depending on the species of the evaluator (*F. sanguinea* or *F. fusca*). The *letters above ants* denote the species identity: “s”—*F. sanguinea*, “f”—*F. fusca*, “p”—*F. polyctena*. *Dashed arrows* connect the stock colony to daughter ones. Note that “Mixed queenright *F. sanguinea* colony” could play the role of “Alien queenright *F. sanguinea* colony” when the evaluators came from one of the other seven remaining colonies

We examined discriminatory abilities of *F. sanguinea* ants from the queenright colonies and their slaves. These ants played the role of evaluators in behavioral tests. They were exposed to the contact with conspecific or allospecific cue-bearers from other queenright colonies. Moreover, we examined the reaction of *F. sanguinea* evaluators and their slaves toward *F. fusca* ants from free-living (queenright) colonies and toward *F. polyctena* ants, the latter to be treated as a positive control, i.e. ants which were expected to elicit the highest level of aggression. As a negative control, ants from the same colony were used. In the separation experiment, evaluators were subjected to the presence of cue-bearers from pure or mixed daughter colonies. Altogether, taking into consideration the category of cue-bearer and species of the evaluator (*F. sanguinea* or *F. fusca*), behavioral tests were performed in 20 treatment combinations, four of which were negative controls (nestmates) (Fig. 1). Each treatment was tested in 3 replications per colony for a total of 24 tests per treatment. In the case of one colony, this rule had to be broken since it contained too few slaves to carry out the planned experimental design. These missing tests were carried out using slaves from other colonies. Tests in which no interaction occurred (*n* = 3) were excluded from further analysis. Behavioral bioassays were performed between July 2–22, 2014 in randomized order to avoid bias related to time or a regular sequence of treatments.

### Statistical analyses

The frequency of pooled behaviors (i.e. antennation and aggression) was explained by treatment combinations after fitting the generalized linear mixed model with the negative binomial distribution of the dependent variable to address count data over-dispersion, with evaluator ants’ colony of origin as a random effect. A similar model was applied to the data set on the frequency of antennation acts only, except that the distribution of the dependent variable was defined to follow the Poisson process. The analysis of aggressive behaviors had to handle many zero values in some treatments (meaning no aggression during the tests) and positive-only in the others. Such extreme differences in outcome of behavioral tests between the treatments made it difficult to fit one model for full data on aggressive behaviors. Therefore, we applied two separate models for simplified or reduced datasets and also made some indirect inferences based on the comparison of the results between model on all behaviors and antennation only. The logistic binary regression with colony-level random term was applied after the binary coding of the response variable, which meant the assignment of the value of zero to the tests in which no aggressive behavior was recorded, and the value of one to the tests with a non-zero (positive) frequency of aggressive behaviors. If all tests representing a given treatment obtained only zero or only one values, they had to be excluded from the analysis due to problems with a reliable estimation of the model parameters in such cases. The post hoc Tukey procedure was used to find significant differences in pair-wise comparisons between treatments. Moreover, we wanted to determine whether our experimental treatments could explain the variance in the intensity of an aggressive response among behavioral tests as measured by the frequency of aggressive acts, taking into account those tests in which a value of one was assigned in the binary coding. Consequently, the generalized linear mixed model was fitted with a zero-truncated Poisson distribution of the response variable, with the colony as a random effect. An additional observation-level random term was introduced to account for data over-dispersion. This model was built using a MCMCglmm package for R which applies Markov chain Monte Carlo technique for the simulation of posterior distributions of the model parameters (Hadfield 2010). Estimates of regression coefficients for each treatment were obtained after removal of the intercept. Significant differences between treatments were determined based on the highest posterior density intervals for the posterior means. Treatments in which aggressive behavior was recorded in less than 6 tests were excluded from this analysis because the sample size was too low. The subset of the most aggressive behaviors (i.e. biting with flexed gaster) was compared among the six treatments by fitting the linear mixed model after square-root data transformation. Statistical computations were performed with the use of SAS 9.3 and R (packages lme4 v. 1.1-7, multcomp v. 1.3-8, MCMCglmm v. 2.21, coda v. 0.16-1) software (Plummer et al. 2006; Hothorn 2008; Hadfield 2010; Bates et al. 2014; R Development Core Team 2015).

## Results

### Antennation

*Formica fusca* displayed a significantly higher frequency of antennal contacts toward *F. sanguinea* ants from alien queenright colonies than toward the following categories of cue-bearers: conspecific nestmates (*t* = 4.31, *p* = 0.002), former *F. fusca* nestmates from mixed (bispecific) and species-separated groups (*t* = 5.01, *p* < 0.001; *t* = 4.62, *p* < 0.001, respectively), former *F. sanguinea* nestmates from mixed groups (*t* = 3.36, *p* < 0.039), and *F. polyctena* ants (*t* = 4.45, *p* = 0.001). In tests where *F. sanguinea* ants were used as evaluators, *F. polyctena* ants received antennal contacts less frequently than *F. sanguinea* and *F. fusca* ants from alien colonies (*t* = −3.42, *p* = 0.032; *t* = −4.39, *p* = 0.002, respectively), as well as *F. sanguinea* and *F. fusca* former nestmates from mixed groups (*t* = −3.98, *p* = 0.006; *t* = −3.56, *p* = 0.022, respectively).

### Discrimination of non-nestmates from queenright parasitic colonies

**Fig. 2:**
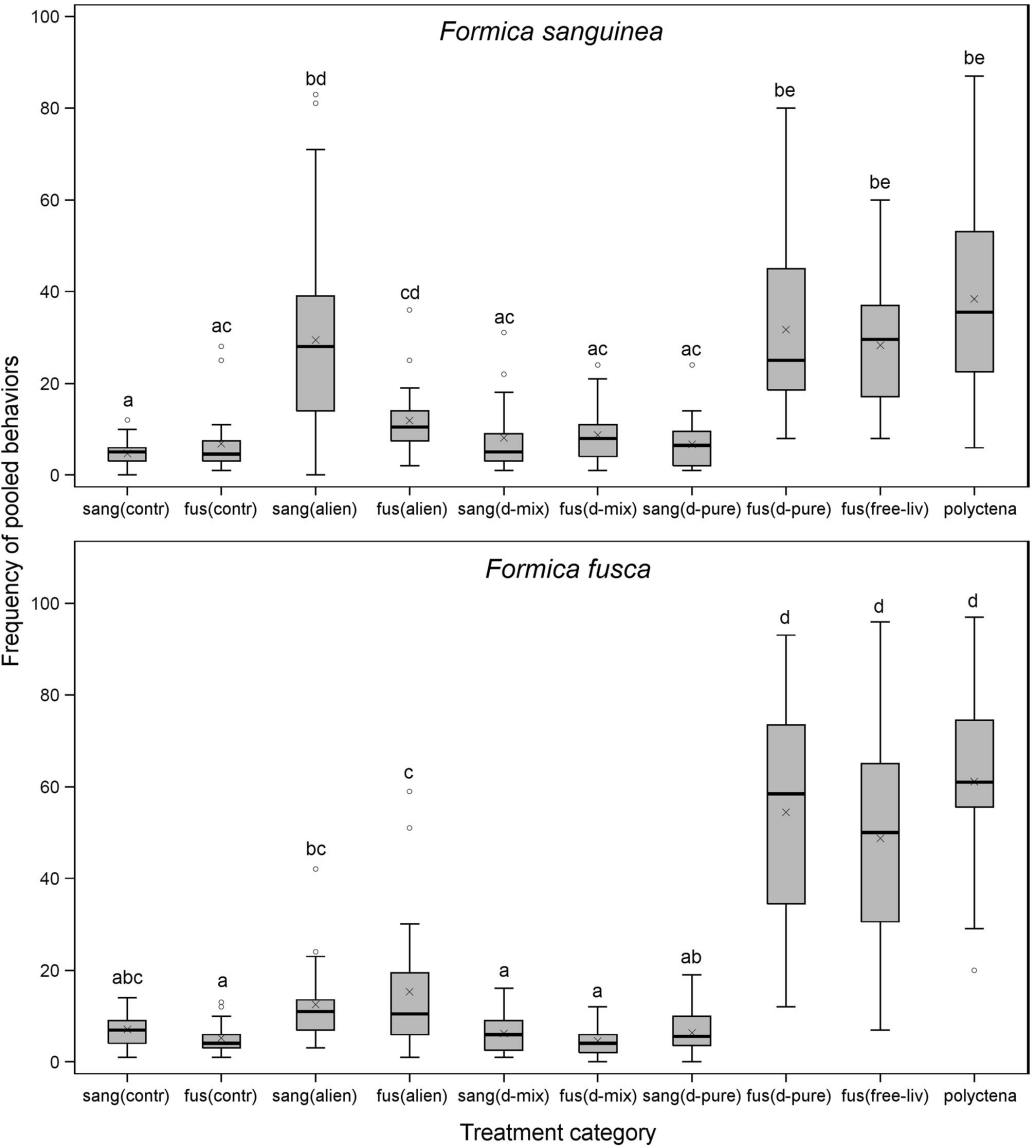
Comparison of frequencies of pooled behaviors (i.e. antennation and aggression) displayed by *F. sanguinea* and *F. fusca* ants toward different categories of cue-bearers during behavioral tests. *Each bar* covers the range of observed values between the first and third quartile with the inner line indicating the median and the “*x*” symbol—mean value. *Whiskers* depict minimal and maximal values, excluding outliers, which are marked separately. Treatments sharing a *common letter* within the same species of evaluator are not different at *p* value <0.05 (after Tukey’s correction for multiple comparisons following the generalized linear mixed model procedure using negative-binomial distribution). Sample size for each treatment is given in Table 1. The explanation of the abbreviations of treatment categories is given in Fig. 1

**Fig. 3:**
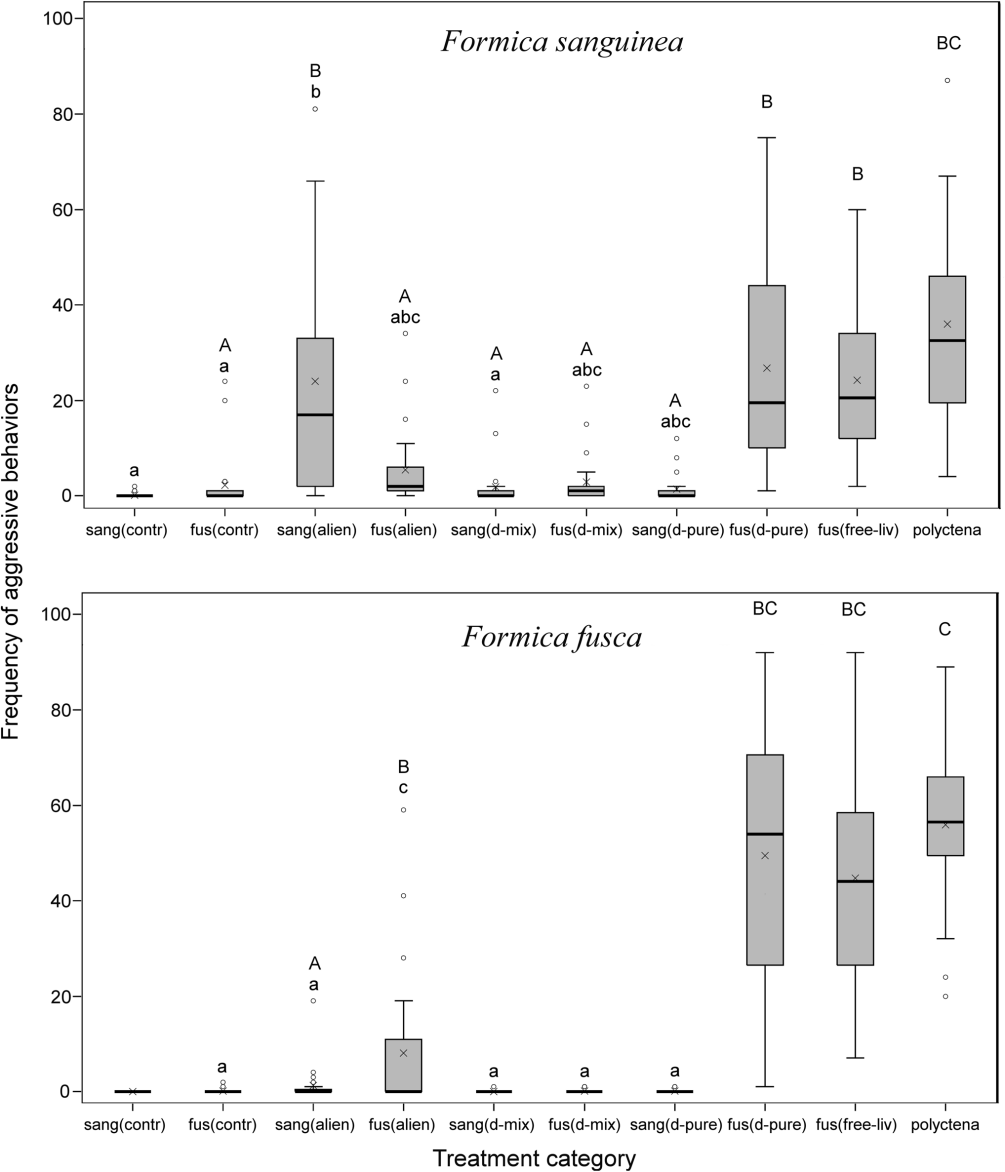
Overview of aggressive responses of *F. sanguinea* and *F. fusca* evaluators to ants representing different treatment categories. *Each bar* covers the range of observed values between the first and third quartile with the inner line indicating the median and the “*x*” symbol—mean value. *Whiskers* depict minimal and maximal values, excluding outliers, which are marked separately. Treatments sharing a *common capital letter* are not different in the model on the intensity of aggressive response (after Bonferroni correction for multiple comparisons). Those which share *common lower case letter* are not different in the model on the presence/absence of aggression among behavioral tests (after Tukey’s correction for multiple comparisons following logistic binary regression). *No letter* of a given case means that the treatment has not been included in respective model. The explanation of the abbreviations of treatment categories is given in Fig. 1. Results obtained for the model on the intensity of aggressive response are presented in more detail in the online resource (Table S1)

The aggressive reaction of both *F. sanguinea* and their slaves proved to be more intense toward conspecific non-nestmates from alien parasitic colonies than toward allospecific ones (Fig. 3). In accordance with this result, ants of either species targeted more behavioral acts toward alien conspecific ants than toward nestmates (Fig. 2). Moreover, the frequency of tests in which *F. sanguinea* ants exhibited aggressive behavior was higher when a slave-making ant or a slave from an alien parasitic colony was presented, compared to respective control treatments with use of a nestmate ant (Fig. 3). The intensity of the aggressive behavior of *F. fusca* evaluators toward alien slaves did not differ from that toward *F. fusca* ants from free living-colonies (Fig. 3; Table S1). However, note that in the alien slave treatment, the aggressive behavior of *F. fusca* ants was recorded in only 37.5 % of tests, which translated into a significantly lower frequency of behaviors in the model of pooled data (Table 1; Fig. 2). In the case of *F. sanguinea* ants, the frequency of aggressive behaviors and pooled behaviors targeted at conspecific non-nestmates did not differ from those targeted at allospecific competitors (*F. polyctena*) (Fig. 2; for pooled behaviors: *t* = −0.11, adjusted *p* value = 1). However, the difference is clear when only the most aggressive behaviors (i.e. biting with flexed gaster) are taken into consideration, since they were almost never observed to be displayed by *F. sanguinea* ants toward conspecific individuals (*p* value in Wilcoxon signed rank test for data averaged for each colony <0.01, *n* = 8).

**Table 1:** Sample size for each treatment along with number of behavioral tests and colonies in which aggression has been recorded

Species of evaluator	Treatment category	Total number of tests	Occurrence of aggression	
			Number of tests	Number of colonies (max. 8)
*Formica sanguinea*	sang(contr)	23	3	3
	fus(contr)	24	8	5
	sang(alien)	24	22	8
	fus(alien)	23	19	8
	sang(d-mix)	24	7	5
	fus(d-mix)	23	12	7
	sang(d-pure)	24	10	6
	fus(d-pure)	24	24	8
	fus(free-liv)	24	24	8
	polyctena	24	24	8
*Formica fusca*	sang(contr)	25	0	0
	fus(contr)	25	2	2
	sang(alien)	24	7	5
	fus(alien)	24	9	5
	sang(d-mix)	24	1	1
	fus(d-mix)	24	2	2
	sang(d-pure)	21	2	2
	fus(d-pure)	24	24	8
	fus(free-liv)	24	24	8
	polyctena	24	24	8

The explanation of the abbreviations of treatment categories is given in Fig. 1

### Aggression toward free-living *F. fusca*

**Fig. 4:**
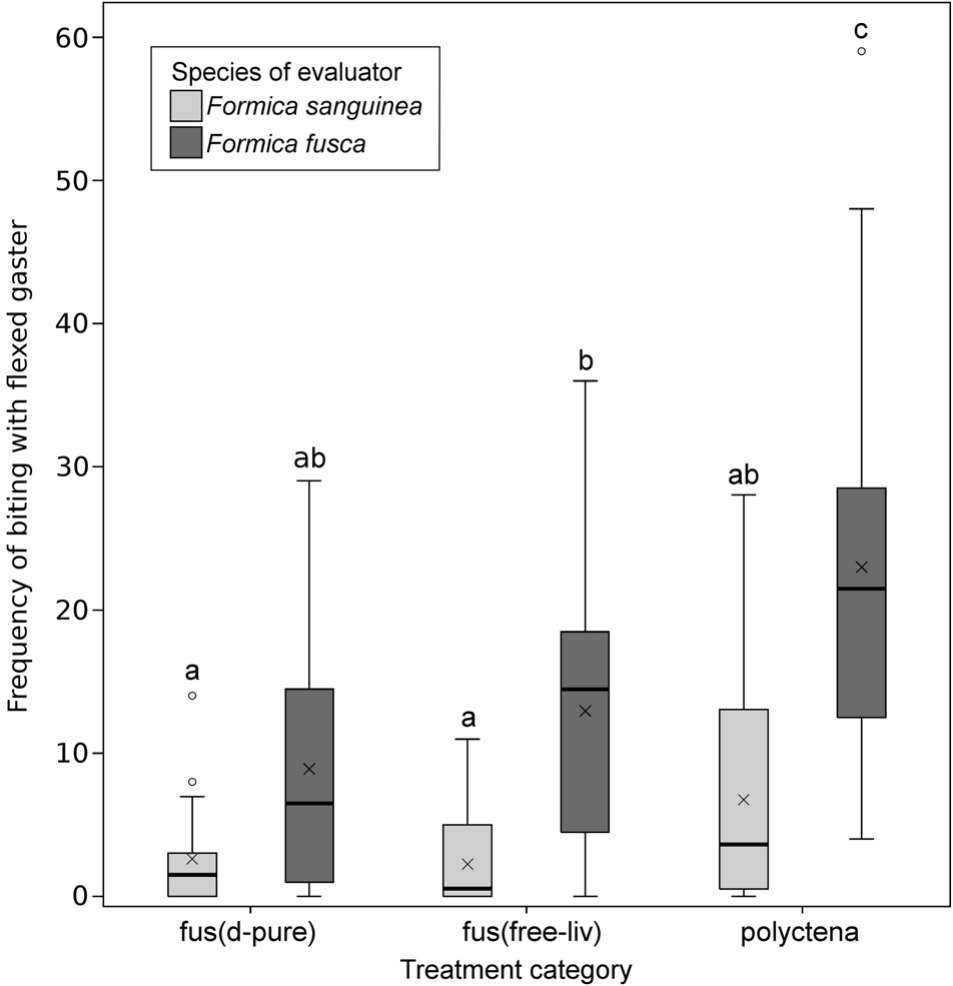
Frequency of the most aggressive behaviors (biting with flexed gaster) in six treatments characterized by high overall aggression. *Each bar* covers the range of observed values between the first and third quartile with the inner line indicating the median and the “*x*” symbol—mean value. *Whiskers* depict minimal and maximal values, excluding outliers, which are marked separately. *Different letters above the bars* indicate statistically significant differences between treatments (Tukey post hoc comparisons following the linear mixed model on square-root transformed data)

Both *F. sanguinea* and their slaves exhibited a higher frequency of pooled behaviors toward *F. fusca* ants from free-living colonies than toward nestmates (*p* values <10^−4^; Fig. 2). This effect can be attributed to aggressive behaviors only, since the model on antennation resulted in higher least square means for control treatments. Moreover, the slaves displayed more behaviors toward free-living *F. fusca* than toward ants from alien parasitic colonies (Fig. 3). Again, this difference can be attributed to different levels of aggression, since the analysis on the frequencies of antennations showed a reverse tendency, although not significant. Furthermore, *F. sanguinea* behaved in an aggressive manner toward free-living *F. fusca* more often than toward alien slaves, which translated into significant differences between the respective treatments in the analysis on combined behaviors as well as on aggressive ones only (Figs. 2, 3; Table S1 ). On the other hand, no such differences were detected between treatments in which free-living *F. fusca* or *F. sanguinea* ants from alien colonies were used as cue-bearers, showing a high level of aggression of slave-making ants in both those combinations (Fig. 3). The frequency of aggressive acts targeted at free-living *F. fusca* displayed by both the parasite and its slaves did not differ from that directed against *F. polyctena* individuals. However, *F. fusca* ants displayed a higher frequency of biting with flexed gaster toward *F. polyctena* than toward conspecific ants from the free-living colony (Fig. 4).

### Effect of species-separation

The separation of mixed-species groups from the stock colony did not cause any significant change in the behavior of former nestmates toward ants treated in this way. This result shows that the separation *per se* had not influenced significantly recognition labels of ants. Similarly, ants of both species apparently recognized *F. sanguinea* individuals, which had been reared in pure colonies, as nestmates (Figs. 2, 3). In contrast, species-separation affected *F. fusca* slaves in a manner which caused rejection and hostility exhibited by former nestmates (Figs. 2, 3; Table S1). The lack of contact with slave-makers had apparently changed the recognition label of *F. fusca* ants from pure colonies to such a degree that they did not differ in the frequency of provoked aggressive behaviors from conspecific ants originating from free-living colonies.

## Discussion

The pattern of aggression differences in bioassays confirms our hypothesis (3) that *F. sanguinea* ants exert an impact on the recognition signature of their *F. fusca* slaves. This conclusion is supported also by the results of chemical analyses which have demonstrated that CHC profile of enslaved *F. fusca* ants is enriched with heavier CHC fraction present on slave-makers’ cuticle but absent from CHC profile of *F. fusca* ants from free-living colonies (Włodarczyk and Szczepaniak in prep.). On the other hand, it seems that the change in the recognition label of *F. sanguinea* ants from a species-separated colony was too small to elicit the aggression of former nestmates from the stock colony. It cannot be ruled out that such aggression could have eventually appeared after a longer isolation period due to the continuous loss of heterospecific recognition cues (Errard 1994b). Nonetheless, our results support the hypothesis that *F. sanguinea* ants reduce within-colony species-related differences in recognition signature by the promotion of own recognition cues which are transferred to slaves. Obviously, the effectiveness of this strategy is dependent on the actual colony’s ratio of parasites to slaves (see Włodarczyk and Szczepaniak 2014). The importance of the parasite-to-slave transfer of recognition cues for colony integrity is highlighted by the fact that a period of less than 2 months was long enough for *F. fusca* ants to be treated by former nestmates with similar hostility as *F. fusca* ants from free-living colonies.

*Formica sanguinea* ants discriminated slave-making ants from alien queenright colonies better than their slaves. Moreover, the intensity of this response was comparable to that directed against the allospecific competitor—*F. polyctena*. This result can be explained by the development of a more accurate template by the parasitic ants. Such a trait may have evolved as an adaptation for efficient nestmate recognition under conditions of a high diversity of reference odors within the colony. In the case of slaves, the same conditions seemingly caused the template developed during odor learning to be broad enough to allow false acceptances. It has been shown that within-colony recognition cue diversity may reduce the discriminatory abilities of ants, possibly due to the formation of a broader or generalized template (Tsutsui et al. 2003; Errard et al. 2006a). Similar phenomenon was observed in *Polistes biglumis* paper wasps which made more recognition errors if they colonies were parasitized by *P. atrimandibularis* (Lorenzi 2003). The latter species is an inquiline and its CHC profile includes alkenes during most part of its life cycle, whereas this class of compounds is almost absent on the host’s cuticle (Bagnères et al. 1996). However, in *P. biglumis* recognition errors included also false rejection which apparently did not occurred in our study. Nevertheless, it can not be ruled out that possibility of detection of such errors was limited by our experimental set-up and that they are indeed made by slaves toward newly eclosed, unrelated slaves.

Although some results presented here can be attributed to the effect of high within-colony recognition cues variability, slave ants have not lost their ability to discriminate conspecific ants from free-living colonies. The high aggression displayed toward them, no different in terms of the frequency of aggressive acts from aggression exhibited toward *F. polyctena,* is consistent with the observed CHC differences between enslaved and free-living *F. fusca* (Włodarczyk and Szczepaniak in prep.). However, if we concentrate on the frequency of biting with flexed gaster, which can be considered the most aggressive response, it appears that the allospecific competitor *F. polyctena* is treated by slaves with greater hostility than conspecific ants from free-living colonies. Analogously, the intensity of the aggressive response of *F. sanguinea* evaluators was no different between the treatment groups in which alien conspecifics or *F. polyctena* were presented as cue-bearers. Nevertheless, the frequency of biting with flexed gaster shows a lower level of aggression toward conspecific competitors. This effect can be explained by the greater similarity between conspecific individuals, whose recognition errors are embedded in trade-offs between the costs of false acceptances and rejections (Reeve 1989; Stuart 1991). Another not mutually exclusive explanation is that both studied species minimize the costs of competition by reducing intercolonial aggression. At the extreme, such a strategy is represented by sophisticated forms of ritualization in intraspecific conflicts (Hölldobler 1981; Czechowski 1984). The observations of Czechowski (1994a) are consistent with our results, as he found that offering to *F. sanguinea* colonies a large number of *F. polyctena* pupae, which became adopted and developed into adult workers, was followed by the aggression of *F. sanguinea* ants from alien colonies. Such a change in intraspecific relations was seemingly caused by the recognition label of *F. sanguinea*, which had shifted toward that characteristic for *F. polyctena* (Czechowski 1994b; Włodarczyk 2012; Włodarczyk and Szczepaniak 2014).

It can also be argued that the low aggression of *F. fusca* ants toward ants from alien parasitic colonies results from the fact that they were, unlike *F. sanguinea*, sampled from inside the nest. Tasks inside the nest, as safer, are expected to be performed by younger workers, which have a longer life expectancy and consequently are less apt to undertake risky behavior (Jeane 1986; Tofilski 2002). However, our colonies were collected from the field in the spring, which is before the period of raiding activity of *F. sanguinea* colonies. Thus, the slaves used in our experiment were not young individuals, since they had hatched from pupae robbed in the previous year or earlier. Moreover, task- or age-related differences in aggression should be independent of the type of threat (Norman et al. 2014). We did not find evidence for a difference in the general aggression between slave-making ants and their slaves, since ants of both these species responded in a highly aggressive way toward *F. polyctena* ants (positive control) and remained non-aggressive toward nestmates (negative control), indicating that the application of different sampling method for each species did not affect our results.

When we solely analyze the proportion of tests in which aggressive responses occurred, there is no evidence that *F. fusca* slaves discriminated between ants of different species from alien parasitic colonies. However, a comparison of the intensity of aggressive responses reveals that it is the slave species which elicits higher aggression in *F. fusca* evaluators. A reverse tendency has been observed for *F. sanguinea*, which exhibited lower aggression toward alien slaves than toward alien conspecific ants. This difference can be explained by one of our hypotheses. Namely, we propose that the pattern of discriminatory of *F. sanguinea* ants toward individuals from alien parasitic colonies results from the fact that this slave-making species specifically recognizes the slaves and displays an intrinsic tolerance of them. Such tolerance might be an adaptation for the easier acceptance of new slaves originating from a colony not exploited in the recent past. These slaves might possess their own distinct odor variant owing to the remarkable inter-colonial differences in the CHC profiles of *F. fusca* (Martin et al. 2011). Slave-makers displayed aggressive behavior toward alien slaves in a higher proportion of tests than they did toward their own slaves, hence they detected the dissimilarity of recognition label of ants from alien colonies. However, the postulated intrinsic tolerance exhibited by of *F. sanguinea* ants caused the intensity of the aggressive response to alien slaves to be relatively low, especially if we compare it to the behavior observed in the equivalent situation with *F. fusca* evaluators. The lack of overt aggression toward alien slaves has also been observed in field conditions, during a conflict between *F. sanguinea* colonies (Czechowski 1990). The hypothesis on broader tolerance toward individuals which are likely to be slaves also explains why non-nestmate slaves, as opposed to their parasites, were accepted by *F. sanguinea* evaluators. Otherwise, the alien slaves should be rejected, as can be concluded from the results of the separation experiment showing that slaves had acquired nestmate recognition cues from *F. sanguinea* ants, which themselves elicited aggression. What is more, the incorporation of parasite-derived components into their own recognition signature is necessary for *F. fusca* individuals to be recognized as slaves. The species-specific recognition signature of *F. fusca* ants, unless having been effectively modified by a parasite, does not induce the tolerance of *F. sanguinea*, as can be concluded from the high rate of aggressive behaviors toward *F. fusca* ants from species-separated and free-living colonies. These conclusions suggest the involvement of a neural mechanism other than the simple desensitization of CHC receptors during the recognition of *F. fusca* slaves. It is more likely that information on the CHC profile of slaves is processed in higher brain centers, for instance, in mushroom bodies (Ozaki and Hefetz 2014).

Differences between *F. sanguinea* and their slaves in the pattern of discriminatory behavior toward individuals from alien mixed colonies can alternatively be explained without invoking the adaptation to a parasitic mode of life. It is possible that each species differs in sensitivity to particular CHCs or in the algorithm used to assess dissimilarity. Furthermore, the hydrocarbons specific for *F. sanguinea* might lose their usefulness as recognition cues for this species after being diluted in the blend already present on the *F. fusca* cuticle, thus explaining the low level of aggression toward alien slaves. Correspondingly, the relatively low ability of *F. fusca* ants to discriminate conspecific individuals from alien parasitic colonies may be a consequence of greater variability of *fusca*-derived cues in a mixed colony, which makes them harder to serve as colony-specific signals. Thus, additional studies are needed for the confirmation of our hypotheses (1) and (2).

## Electronic supplementary material

Below is the link to the electronic supplementary material.

Supplementary material 1 (DOC 83 kb)
