# Supplementary material for: Discriminatory abilities of facultative slave-making ants and their slaves
Source: Insectes Soc. 2016 Jun 17;63(4):507–17. doi: 10.1007/s00040-016-0493-z (PMC5052306; doi:10.1007/s00040-016-0493-z)
Supplement: Supplementary file 1 — Supplementary material 1 (DOC 83 kb) [file 40_2016_493_MOESM1_ESM.doc]

Table S1. Results of pairwise comparisons among treatments calculated from the output of the MCMC simulation applied to the generalized linear mixed model with zero-truncated Poisson distribution. The values indicate the posterior mean of the regression coefficient for a treatment given in the row minus the that of a treatment in the column. Significant differences are marked with asterisks: * *p* < 0.05, ** *p* < 10^-2^, *** *p* < 10^-3^, **** *p* < 10^-4^, ***** *p* < 10^-5^

| Species of evaluator | | *F. sanguinea* | | | | | | | | |  | *F. fusca* | | | | |
| --- | --- | --- | --- | --- | --- | --- | --- | --- | --- | --- | --- | --- | --- | --- | --- | --- |
|  | Treatment category | fus(contr) | sang(alien) | fus(alien) | sang(d-mix) | fus(d-mix) | sang(d-pure) | fus(d-pure) | fus(free-liv) | polyctena |  | sang(alien) | fus(alien) | fus(d-pure) | fus(free-liv) | polyctena |
| *F. sanguinea* | fus(contr) | - | **-1.72** | -0.17 | 0.06 | 0.01 | 0.65 | **-1.84** | **-1.89** | **-2.3** |  | -0.01 | **-1.58** | **-2.60** | **-2.54** | **-2.85** |
|  |  |  | ****** |  |  |  |  | ****** | ****** | ******** |  |  | ***** | ******** | ******** | ******** |
|  | sang(alien) | **1.72** | - | **1.56** | **1.78** | **1.73** | **2.37** | -0.12 | -0.16 | -0.58 |  | **1.71** | 0.14 | -0.88 | -0.82 | **-1.13** |
|  |  | ****** |  | ****** | ****** | ****** | ******* |  |  |  |  | ***** |  |  |  | ***** |
|  | fus(alien) | 0.17 | **-1.56** | - | 0.23 | 0.17 | 0.82 | **-1.67** | **-1.72** | **-2.14** |  | 0.16 | **-1.41** | **-2.43** | **-2.37** | **-2.69** |
|  |  |  | ****** |  |  |  |  | ****** | ****** | ******** |  |  | ***** | ******4** | ******** | ******** |
|  | sang(d-mix) | -0.06 | **-1.78** | -0.23 | - | -0.06 | 0.59 | **-1.9** | **-1.95** | **-2.36** |  | -0.07 | **-1.64** | **-2.66** | **-2.6** | **-2.91** |
|  |  |  | ****** |  |  |  |  | ****** | ****** | ******** |  |  | ***** | ******** | ******-4** | ******** |
|  | fus(d-mix) | -0.01 | **-1.73** | -0.17 | 0.06 | - | 0.65 | **-1.84** | **-1.89** | **-2.31** |  | -0.01 | **-1.59** | **-2.6** | **-2.54** | **-2.86** |
|  |  |  | ****** |  |  |  |  | ****** | ****** | ******** |  |  | ***** | ******** | ******** | ******** |
|  | sang(d-pure) | -0.65 | **-2.37** | -0.82 | -0.59 | -0.65 | - | **-2.49** | **-2.54** | **-2.95** |  | -0.66 | **-2.23** | **-3.25** | **-3.19** | **-3.50** |
|  |  |  | ******* |  |  |  |  | ******* | ******* | ******** |  |  | ****** | ******** | ******** | ********* |
|  | fus(d-pure) | **1.84** | 0.12 | **1.67** | **1.9** | **1.84** | **2.49** | - | -0.05 | -0.46 |  | **1.83** | 0.26 | -0.76 | -0.7 | **-1.01** |
|  |  | ****** |  | ****** | ****** | ****** | ******* |  |  |  |  | ****** |  |  |  | ***** |
|  | fus(free-liv) | **1.89** | 0.16 | **1.72** | **1.95** | **1.89** | **2.54** | 0.05 | - | -0.42 |  | **1.88** | 0.31 | -0.71 | -0.65 | **-0.97** |
|  |  | ****** |  | ****** | ****** | ****** | ******* |  |  |  |  | ****** |  |  |  | ***** |
|  | polyctena | **2.30** | 0.58 | **2.14** | **2.36** | **2.31** | **2.95** | 0.46 | 0.42 | - |  | **2.30** | 0.72 | -0.30 | -0.24 | -0.55 |
|  |  | ******** |  | ******** | ******** | ******** | ******** |  |  |  |  | ****** |  |  |  |  |
| *F. fusca* | sang(alien) | 0.01 | **-1.71** | -0.16 | 0.07 | 0.01 | 0.66 | **-1.83** | **-1.88** | **-2.30** |  | - | **-1.57** | **-2.59** | **-2.53** | **-2.85** |
|  |  |  | ***** |  |  |  |  | ****** | ****** | ****** |  |  | ***** | ******** | ******** | ******** |
|  | fus(alien) | **1.58** | -0.14 | **1.41** | **1.64** | **1.59** | **2.23** | -0.26 | -0.31 | -0.72 |  | **1.57** | - | -1.02 | -0.96 | **-1.27** |
|  |  | ***** |  | ***** | ***** | ***** | ****** |  |  |  |  | ***** |  |  |  | ***** |
|  | fus(d-pure) | **2.6** | 0.88 | **2.43** | **2.66** | **2.60** | **3.25** | 0.76 | 0.71 | 0.30 |  | **2.59** | 1.02 | - | 0.05 | -0.26 |
|  |  | ******** |  | ******** | ******** | ******** | ******** |  |  |  |  | ******** |  |  |  |  |
|  | fus(free-liv) | **2.54** | 0.82 | **2.37** | **2.6** | **2.54** | **3.19** | 0.70 | 0.65 | 0.24 |  | **2.53** | 0.96 | -0.06 | - | -0.31 |
|  |  | ******** |  | ******** | ******** | ******** | ******** |  |  |  |  | ******** |  |  |  |  |
|  | polyctena | **2.85** | **1.13** | **2.69** | **2.91** | **2.86** | **3.50** | **1.01** | **0.97** | 0.55 |  | **2.85** | **1.27** | 0.26 | 0.31 | - |
|  |  | ******** | ***** | ******** | ******** | ******** | ********* | ***** | ***** |  |  | ******** | ***** |  |  |  |
